# Extreme heat: a global call to action

**DOI:** 10.2471/BLT.25.293342

**Published:** 2025-08-01

**Authors:** Ankur Rakesh, Rajesh Sreedharan, Joy Shumake-Guillemot, Daniela Jacob, Virginia Murray, Kristie Ebi

**Affiliations:** a Health Security and Preparedness, Country Capacity Assessment and Planning Unit, WHO Health Emergencies Programme, World Health Organization, Avenue Appia 20, 1211 Geneva 27, Switzerland.; b Joint Climate and Health Office, World Meteorological Organization, Geneva, Switzerland.; c Climate Service Center Germany, Helmholtz-Zentrum Hereon, Hamburg, Germany.; d Global Disaster Risk Reduction, UK Health Security Agency, London, England.; e Center for Health and the Global Environment, University of Washington, Seattle, United States of America.

In July 2024, the United Nations (UN) Secretary-General issued the Call to Action on Extreme Heat, emphasizing the increasing threat posed by more frequent, intense and longer heatwaves.^1^ He called on governments, policy-makers and the health sector to unite in addressing this crisis, mobilizing efforts to protect vulnerable populations and limit global temperature rise to 1.5 °C above preindustrial temperatures. As global temperatures continue to climb, heatwaves are among the most visible and deadly climate-health emergencies.^2^ The top 10 hottest years on record all occurred in the past decade.^3^ The year 2024 saw record-breaking temperatures across Europe, North America and Asia, exposing populations to extreme conditions that overwhelmed health systems.^4^^,^^5^ The impacts of extreme heat disproportionately affect the most vulnerable, including older people, pregnant women, children, people with chronic illnesses, outdoor workers and socioeconomically disadvantaged communities – particularly in urban areas with inadequate infrastructure and limited access to essential services including health care, water and electricity.^2^^,^^5^

The UN Secretary-General emphasized the critical need for comprehensive and effective heat action plans that incorporate early warning systems based on local meteorological and health data, and identify high-risk populations and areas as well as strategies to strengthen social safety nets and infrastructure.^1^ Some countries are developing national heat action plans that focus broadly on societal adaptation to heat through infrastructure, energy and urban planning. Health-centric plans to specifically address the immediate and long-term effects of heat on human health are needed.^6^ Such planning is most effective when it is part of a broader, cross-sectoral coordinated collaboration based on risk and capacity assessments that explicitly consider health security and preparedness, including factors such as geographic location, occupational and housing conditions, and socioeconomic status to protect the most at-risk communities.^7^ Integrating heat action plans into national capacity development plans such as the national action plans for health security would be helpful to increase the effectiveness of planning. These plans are a country-owned, multi-year planning process that can accelerate the implementation of actions to address extreme heat. 

To build institutional capacity to respond swiftly and effectively to heat crises as part of national preparedness to health emergencies, governments must formally recognize these crises as a public health hazard within their national health plans.^5^

With limited progress in implementing heat action plans following the UN Secretary-General’s call, health systems remain unprepared. This implementation gap could be addressed if governments integrate their heat action plans into national health plans, policies and strategies. Doing so would promote multihazard preparedness and facilitate technical and financial support and cross-sectoral collaboration; it would also institutionalize climate–health integration in alignment with international frameworks. This integration should be noted during national International Health Regulations (2005) capacity assessments, such as the mandatory States Parties Annual Reporting and the Joint External Evaluations. Subsequently, governments could consider these assessment findings when developing their national health plans such as the national action plans for health security, using the clearly defined hazard classification for extreme heat.^5^^,^^8^^,^^9^

Priority actions include strengthening public health capacities for surveillance, risk assessment and early warning systems; enhancing risk communication and community engagement strategies; reinforcing the health workforce and infrastructure; improving access to medical countermeasures; and promoting cross-sectoral collaboration and resource mobilization. All these actions would foster coordinated preventive action across sectors.^5^^,^^10^

To be effective, all relevant plans must be fully resourced financially and technically, which requires substantial investments in health system strengthening and resilience-building measures in addition to nurturing a global pool of experts with experience in managing extreme heat risks. Health ministries should work closely with international organizations, such as the World Health Organization, the International Labour Organization, International Federation of Red Cross and Red Crescent Societies, United Nations Children’s Fund and World Meteorological Organization, to secure the necessary funding and technical assistance to ensure health systems are prepared for the multipronged threats of extreme heat. Ensuring equity is central to these plans.

The UN’s Call to Action on Extreme Heat is an urgent appeal for governments, policy-makers and the health sector to address the profound threat climate change presents to health and well-being. Governments must implement equitable, evidence-informed heat action plans, strengthen health systems and reduce greenhouse gas emissions. Our planet’s future, and the lives of millions, depend on our collective ability to respond to the challenge of rising temperatures.^1^
